# Verification of a 6-part differential haematology analyser Siemens Advia 2120i

**DOI:** 10.11613/BM.2022.020710

**Published:** 2022-06-15

**Authors:** Helena Čičak, Vanja Radišić Biljak, Ana-Maria Šimundić

**Affiliations:** 1 Department of Medical Laboratory Diagnostics, University Hospital “Sveti Duh”, Zagreb, Croatia; 2 Faculty of Pharmacy and Biochemistry, University of Zagreb, Zagreb, Croatia

**Keywords:** verification, haematology analyser, haematology, blood cell count

## Abstract

**Introduction:**

The aim of this study was to perform a comprehensive verification of a 6-part differential haematology analyser Siemens Advia 2120i (Erlangen, Germany), prior to its routine implementation.

**Materials and methods:**

Our verification protocol included: precision (within- and between-run), estimated bias (%) as measure of trueness, which was calculated from observed and manufacturers’ declared value, analytical measuring interval (AMI), carryover, confirmation of a limit of blank (LoB), determination of a limit of detection (LoD) and limit of quantitation (LoQ). The K_2_ ethylenediaminetetraacetic acid (EDTA) patients’ leftover samples were used for verification of analyser Advia 2021i. Acceptance criteria were based on manufacturer technical specifications (Siemens), 2016 state-of-the-art criteria (Vis and Huisman), and EFLM Biological Variation Database.

**Results:**

The within- and between-run precision were acceptable for all parameters and the lowest coefficients of variation were observed for mean corpuscular volume (MCV) (0.3% and 0.6%, respectively). Estimated bias was within the acceptance criteria for all parameters except for MCV (the estimated bias was 2.2% (acceptance criteria 2.0%). AMI was confirmed for all tested parameters (r > 0.99). The carryover estimates ranged from 0.1% for platelet count (Plt) to 0.6% for red blood cell count and were within the manufacturers’ specifications (≤ 1%). Manufacturers’ claims for LoB were confirmed for leukocytes, erythrocytes, haemoglobin, and platelets. The estimated LoD and LoQ were 0.05 x10^9^/L and 0.1 x10^9^/L for white blood cell count, while for Plt values were 2 x10^9^/L and 3 x10^9^/L, respectively.

**Conclusions:**

Analytical performance of the Siemens Advia 2120i meets predefined quality goals and is suitable for routine use in a clinical laboratory.

## Introduction

Haematology analyser (HA) Siemens Advia 2021i (Siemens, Erlangen, Germany) is designed for mid- to high-volume testing in routine work and it measures complete blood count (CBC) with 6-part white blood cell (WBC) differential count (*1*). As HA becoming more technologically advanced and report an extended number of parameters for CBC, it is important to establish all the advantages and limitations of HA by verification.

There are numerous studies that assessed only the precision and/or accuracy as comparability of Siemens Advia 2120i to another type of HA (*2*-*4*). But, estimation of other analytical parameters, such as analytical measuring range (AMI) (linearity), carryover, limit of blank (LoB), limit of detection (LoD) and limit of quantitation (LoQ) in already published articles are fairly scarce. All HA are prone to errors to a more or less extent, and assessment of bias in comparability studies does not reflect the true deviation of measured parameters. As best we know, until now, none of the published studies was verified trueness by reference material (Siemens, Erlangen, Germany) which would enable to estimate the true bias and thus calculate the total error for all the measured parameters. Because of that, we decided for different approach than other studies had for estimation accuracy of our HA.

To the best of our knowledge, none of the studies published until now have performed extended verification of the analytical performance of Advia 2021i prior to implementation in routine work. The aim of this study was to verify the analytical performance of precision, estimate bias as a measure of trueness, AMI (linearity), carryover, LoB, LoD, LoQ and total error (TE) for CBC parameters which measures HA Siemens Advia 2021i.

## Materials and methods

### Study design

This study was performed from April to July 2018 at the Department of Medical Laboratory Diagnostics of University Hospital “Sveti Duh” (Zagreb, Croatia). Verification was performed according to the internally developed protocol, based on Clinical and Laboratory Standards Institute (CLSI) H26-A2, Validation, Verification, and Quality Assurance of Automated Haematology Analysers, CLSI EP17-A Protocols for Determination of Limits of Detection and Limits of Quantification, and International Council for Standardization in Haematology (ICSH) guidelines for the evaluation of blood cell analysers including those used for differential leukocyte and reticulocyte counting (*5*-*7*).

### Materials

For the verification study, the K_2_ ethylenediaminetetraacetic acid (EDTA) (Kima, Piove di Sacco, Italy) patients’ leftover samples were used for. The study had the approval of the hospital Ethics Committee. For estimation of precision and bias, three levels of the commercial ADVIA 3 in 1 TESTpoint Haematology Controls (Siemens, Erlangen, Germany) were used: ABN1 control lot TP81015, NORM control lot TP82015, and ABN2 control lot TP83015. These control samples are haematology reference materials for monitoring the precision and trueness of ADVIA 120/2120/2120i Haematology systems.

### Methods

The analyser Advia 2021i is haematology analyser based on the flow cytometry. Analyser Advia 2021i uses 5 channels to analyse blood samples: i) haemoglobin channel, ii) erythrocyte/platelets channel, iii) peroxidase channel (to distinguish peroxidase-positive cells from peroxidase-negative cells), iv) lobularity/nuclear density channel and v) reticulocyte channel (*8*). Within-run precision was estimated by analysing commercial control samples in a series of 20 replicates and on three patients’ blood samples (10 replicates in a series). Between-run precision was estimated by analysing commercial control samples for consecutive 20 days.

Within-, between-run precision and estimation of bias were determined for leukocytes, erythrocytes, haemoglobin, haematocrit, mean corpuscular volume (MCV), platelets and mean platelet volume (MPV).

Analytical measuring range (linearity) was assessed for leukocytes, erythrocytes, haemoglobin, and platelets of patients’ whole blood samples, with concentrations close to the upper analytical measuring range (linearity) limit. The initial values for leukocytes were 90 x10^9^/L, erythrocytes 7.06 x10^12^/L, haemoglobin 220 g/L, and for platelets 970 x10^9^/L. Based on CLSI H26-A2 guidelines the concept of linearity can only apply to measuring the concentration of haemoglobin, while the term analytical measuring range was used for the measuring number of cells (WBC, RBC, Plt) (*5*). The AMI was verified by preparing serial dilutions of a known high values of certain parameter. Serial dilutions (1:0, 1:2, 1:4, 1:8 and 1:16) were prepared by using the original HA diluent (Sheat rinse ADVIA 2120i, Siemens). Carryover was estimated for leukocytes, erythrocytes, haemoglobin, and platelets by using one sample with low (sample L) and one with high analyte concentration (sample H). Sample H was analysed in triplicate (H1, H2 and H3), followed by the sample L in triplicate (L1, L2 and L3).

The estimation of carryover for each analyte was performed three times, using 3 different H and L samples. Samples with high values had leukocytes > 55.00 x10^9^/L, erythrocytes > 6.00 x10^12^/L, haemoglobin > 170 g/L, and platelets > 720 x10^9^/L. On the other hand, samples with low values had leukocytes < 3.00 x10^9^/L, erythrocytes < 1.10 x10^12^/L, haemoglobin < 20 g/L, and platelets < 25 x10^9^/L. Limit of blank was estimated for leukocytes, erythrocytes, haemoglobin, and platelets by measuring a blank sample (distilled water) in 20 replicates in a series.

Limit of detection was estimated by measuring leukocytes and platelets in six blood samples with very low concentrations of analytes, 10 times in a series. The values of six blood samples for leukocytes were lower than 0.3 x10^9^/L and platelets < 7 x10^9^/L.

Coefficient of variation (CV) was calculated for each sample and for each parameter. For verification of LoQ, six blood samples were analysed with leukocytes values between 0.10-0.17 x10^9^/L and platelets between 2-10 x10^9^/L.

### Statistical analysis

Trueness was estimated by calculating the bias between the mean observed value for every parameter from the between-run precision experiment, and the mean declared value from the manufacturer by using the following equation (Eq.) 1 (*9*):



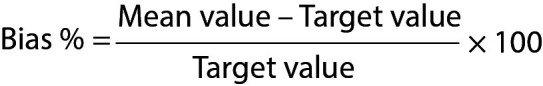



The observed values for verification AMI (linearity) were considered linear if the coefficient of correlation between measured and calculated values was r > 0.99 with P < 0.001. The coefficient of correlation was determined by using MedCalc Statistical Software version 14.8.1 (MedCalc Software bv, Ostend, Belgium).

Carryover was calculated using the following equation (Eq. 2.) (*7*):



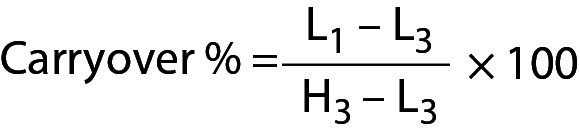



The results were compared with predefined acceptance criteria (criteria from manufacturer and State-of-the-art criteria (SOTA) (*10*). Equation 3. was used for calculating LoB (*6*):



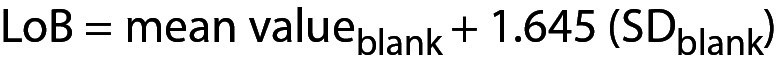



For calculating LoD, equation 4 was used (*6*):



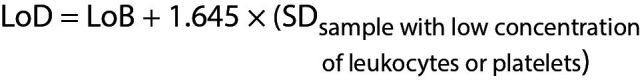



For each sample out of six which was used for verification LoQ, the CV% was determined. The concentration at which CV% was lower than the desired imprecision (15% for leukocytes and 25% for platelets) represented LoQ.

The TE was calculated only for CBC. The TE was calculated according to Eq. 5. (*11*):



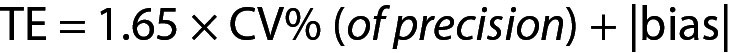



For statistical analysis of obtained data, the Microsoft Office Excel 2010 (Microsoft, Washington, USA) was used. Acceptance criteria for precision, trueness and total error were defined from SOTA 2016 criteria, European Federation of Clinical Chemistry and Laboratory Medicine (EFLM) 2019 Biological Variation Database, and manufacturer technical specifications (*10*, *12*, *13*). Moreover, SOTA performance specifications for haematology parameters was published 2016 by Vis and Huisman (*10*).

## Results

Within-run precision for low concentration of platelets exceeded the SOTA acceptance criterion (4.5%), both in patients’ (7.6%) and control samples (4.8%). The precision of MPV in low concentration control sample was higher that the acceptance criteria both in within-run and between-run precision experiments (4.6 and 4.7% respectively) but was within acceptance criteria in normal and high concentration control samples. Also, within-run precision (1.1%) for low concentrations of Hb (53 g/L) in control samples slightly exceeded from acceptance criteria of the manufacturer and SOTA (0.9%). Other CBC parameters did meet SOTA acceptance criteria, both at normal and high concentration levels, in patients’, as well as in control samples (Table 1).

**Table 1:** Estimated within- and between-run precision for Siemens Advia 2120i haematology analyser

**Paramete**r	**Within-run precision – patients’ samples** **CV% (concentration)**			**Within-run precision – control samples** **CV% (concentration)**			**Acceptance criteria for within-run precision** **CV%**		**Between-run precision control samples** **CV% (concentration)**			**Acceptance criteria for between-run precision** **CV%**		
	L	N	H	L	N	H	Siemens	SOTA	L	N	H	SOTA for values L/N/H	EuBIVAS MIN	EuBIVAS DES
WBC, (x10^9^/L)	4.7 (0.53)	2.0 (6.29)	2.4 (30.48)	2.4 (3.52)	1.6 (7.26)	1.8 (16.84)	2.7	2.5	2.3 (3.45)	2.4 (7.13)	2.5 (16.87)	6.0/2.5/1.5*	8.1	5.4
RBC, (x10^12^/L)	0.8 (2.36)	0.6 (4.15)	0.7 (6.02)	0.7 (2.30)	0.7 (4.32)	0.9 (5.17)	1.2	1.1	0.8 (2.27)	0.8 (4.27)	1.0 (5.15)	1.1	2.0	1.3
Haemoglobin, (g/L)	0.8 (84)	0.6 (124)	0.9 (175)	1.1 (53)	0.7 (112)	0.7 (165)	0.9	0.9	1.7 (54)	1.3 (111)	0.4 (166)	1.0	2.0	1.4
Haematocrit, (L/L)	/	/	/	0.8 (0.17)	0.8 (0.34)	1.0 (0.48)	/	1.2	1.2 (0.16)	1.3 (0.33)	1.2 (0.48)	1.4	2.1	1.4
MCV, (fL)	0.3 (82)	0.3 (91)	0.3 (102)	0.5 (72)	0.3 (79)	0.3 (94)	0.8	0.6	0.7 (71)	0.8 (78)	0.6 (93)	0.8	0.6	0.4
Platelets, (x10^9^/L)	7.6 (20)	2.5 (199)	2.3 (623)	4.8 (74)	2.4 (214)	1.6 (458)	2.9	4.5	4.4 (76)	2.5 (214)	2.2 (463)	4.5/3.0/5.0*	4.2	2.8
MPV, (fL)	/	/	/	4.6 (7.1)	1.2 (7.2)	1.0 (7.1)	/	2.5	4.7 (7.0)	1.5 (7.2)	1.2 (7.1)	2.5	1.7	1.1
Lighter gray - meets at least one of the criteria. Darker gray – did not meet any criteria. *The SOTA criteria define limits of acceptable imprecision for three cut-off values for WBC and platelets. Ranges of values WBC based on SOTA are: L: < 1.0 x10^9^/L, N: 1-10 x10^9^/L and H: > 10 x10^9^/L. Ranges of values platelets based on SOTA are: very low: 10-20 x10^9^/L, low: ~ 50 x10^9^/L and normal: > 50 x10^9^/L. L– low values. N – normal values. H – high values. WBC – White Blood Cell count. RBC – Red Blood Cell count. MCV – Mean Corpuscular Volume. MPV – Mean Platelet Volume. SOTA – State-of-the-Art acceptance criteria. EuBIVAS DES – EFLM Biological Variation Database, Desirable specification. EFLM – European Federation of Clinical Chemistry and Laboratory Medicine.														

The estimated bias exceeded even the SOTA acceptance criteria (2.0%) only for MCV at high concentration control sample (2.2%) (Table 2). The estimated percentage bias was within the acceptance criteria for all other CBC parameters.

**Table 2:** Estimated accuracy on control samples for haematology analyser Siemens Advia 2120i

**Paramete**r	**Bias,%** **(declared concentration by manufacturer)**			**Acceptance criteria,%**		
	L	N	H	SOTA	EuBIVAS MIN	EuBIVAS DES
WBC, (x10^9^/L)	1.1 (3.41)	1.5 (7.02)	1.4 (16.63)	4.4	7.4	4.9
RBC, (x10^12^/L)	-1.7 (2.31)	0.5 (4.25)	-0.4 (5.17)	3.2	2.6	1.8
Haemoglobin, (g/L)	2.1 (53)	1.0 (110)	1.7 (163)	1.3	2.4	1.6
Haematocrit, (L/L)	-0.8 (0.16)	1.8 (0.33)	1.8 (0.47)	1.8	2.3	1.5
MCV, (fL)	0.9 (70)	1.4 (77)	2.2 (91)	2	1.4	0.9
Platelets, (x10^9^/L)	-4.9 (80)	-2.7 (220)	-4.5 (485)	6.4	7.6	5.0
MPV, (fL)	0.1 (7.0)	-0.7 (7.2)	-1.6 (7.2)	/	2.8	1.9
Lighter gray – meets at least one of the criteria. Darker gray – did not meet any criteria. L – low values. N – normal values. H – high values. WBC – White Blood Cell count. RBC – Red Blood Cell count. MCV – Mean Corpuscular Volume. MPV – Mean Platelet Volume. SOTA – State-of-the-Art acceptance criteria. EuBIVAS DES – EFLM Biological Variation Database, Desirable specification. EFLM – European Federation of Clinical Chemistry and Laboratory Medicine.						

There was a linear relationship over the defined analytical range for RBC, WBC, Hb and Plt, with no significant constant and proportional difference. All four parameters showed excellent linearity with a coefficient of correlation 1.00 (95%CI 0.99 to 1.00), P < 0.001 (Supplementary Table 1).

No significant carryover effect was observed for RBC, Hb, WBC and Plt. Estimated carryover was within the manufacturer’s criteria, listed in the Supplementary Table 2. Furthermore, the observed results for leukocytes and haemoglobin for carryover were higher than the SOTA criteria.

Manufacturer’s LoB for leukocytes (< 0.1 x10^9^/L) and for platelets (< 5 x10^9^/L) was verified. The manufacturer did not declare acceptance criteria for erythrocytes and haemoglobin. Furthermore, the obtained results for LoB did meet the SOTA criteria for erythrocytes (< 0.01 x10^12^/L) and haemoglobin (< 0.7 g/L) (data for verification LoB are not shown). LoB Estimated LoD for leukocytes and platelets were 0.05 x10^9^/L and 2 x10^9^/L, respectively. LoQ for leukocytes was estimated at 0.10 x10^9^/L. Estimated LoQ for platelets was 3, even lower than the lower AMI (linearity) limit (Supplementary Table 3.)

The calculated TE for MCV exceeded the acceptance criteria. All other parameters were within the allowable TE (Table 3).

**Table 3:** Estimated precision, bias and total error for Siemens Advia 2120i haematology analyser

**Parameter**	**Between-run precision for control sample N** **CV% (mean concentration)**	**Bias %**	**Total Error**	**Acceptance criteria, %**		
				**EuBIVAS** **OPT**	**EuBIVAS** **DES**	**EuBIVAS** **MIN**
WBC, (x10^9^/L)	2.4 (7.13)	1.5	5.5	6.9	13.8	20.7
RBC, (x10^12^/L)	0.8 (4.27)	0.5	1.8	1.9	3.9	5.8
Haemoglobin, (g/L)	1.3 (111)	1.0	3.5	1.9	3.8	5.8
Haematocrit, (L/L)	1.3 (0.33)	1.9	3.9	1.9	3.9	5.8
MCV, (fL)	0.8 (78)	1.4	2.7	0.8	1.6	2.4
Platelets, (x10^9^/L)	2.5 (214)	-2.7	6.8	4.8	9.7	14.5
MPV, (fL)	1.5 (7.2)	-0.7	3.2	1.9	3.8	5.6
Gray – did not meet any criteria. WBC – White Blood Cell count. RBC – Red Blood Cell count. MCV – Mean Corpuscular Volume. MPV – Mean Platelet Volume. SOTA – State-of-the-Art acceptance criteria. EuBIVAS OPT – EFLM Biological Variation Database, Optimal specification. EuBIVAS DES – EFLM Biological Variation Database, Desirable specification. EuBIVAS MIN – EFLM Biological Variation Database, Minimum specification. EFLM – European Federation of Clinical Chemistry and Laboratory Medicine						

## Discussion

The within- and between-run precision, estimated bias and total error results meet the criteria for all parameters across concentration ranges except for Plt and MCV in low concentrations. The AMI (linearity) was verified, and no significant carryover was observed for RBC, Hb, WBC and Plt.

As previously mentioned, within-run precision for Plt at low concentration did not meet criteria but all laboratory findings with Plt count below 100 x10^9^/L, in our Department are revised microscopically and there is no potential harm for patient due to enlarged within-run CV% for Plt. The SOTA criterion has stricter criteria because carryover of *e.g*., 2% can have a significant impact on results. The largest obtained carryover in this study was for leukocytes and it was 0.8%. For example, if the samples with a higher leukocyte count of 72 x10^9^/L and with a lower count 3 x10^9^/L would be analysed one after the other, and the carryover was 0.8%, the second sample would have falsely increased result of 4 x10^9^/L, which is not clinically significant.

Moreover, obtained results for TE are within desirable the European Biological Variation Study (EuBIVAS) acceptance criteria for all parameters except MCV. The importance of calculating TE is because it describes the precision and estimated bias of certain parameter measurement that can occur. The measurement for certain parameter which do not meet the criteria should be controlled more often and with more consideration of its performance.

Harris *et al.* results agree with ours for within-run precision at certain concentrations which are similar to our normal (N) values for control and patients’ samples (Table 1) (*1*). Also, Harris *et al.* in the other published article only describes technical aspects of Siemens Advia 2021 and states manufacturer claims for analytical measuring range (linearity) of WBC, RBC, Hb and Plt (*8*). In our study we verified this manufacturer’s claims for these parameters and estimated the limit of detection and quantitation for WBC and Plt.

All other studies (*2*-*4*) performed verification of accuracy as comparison and their results showed that the Siemens Advia 2021/2021i was comparable with all other types of HA. In our study, we performed an estimation of bias with reference material and the obtained results implicate that HA Siemens Advia 2021i showed acceptable analytical performance.

As for the limitations of this study, we did not perform validation of morphology flags and calculation of TE for differential blood count nor compare parameters from leukocyte differential count for Advia 2021i with manual differentiation of leukocytes and estimate the diagnostic accuracy of the Advia 2021i with other types of HA. Also, our study has estimated analytical measuring range (linearity), carryover, LoB, LoD and LoQ only for open tube mode because of the limited volume of the samples. The ICSH guidelines for the evaluation of the blood cell analyser advise to assess those analytical performances for both, open and closed tube mode (*7*).

In conclusion, to the best of our knowledge, this study was the first that performed additional analytical performances (linearity, carryover, LoB, LoD, and LoQ) except precision and trueness which were already obtained in other published studies. The HA Siemens Advia 2021i showed reliable analytical performance for standard CBC parameters and it is suitable for routine use in a clinical laboratory.
